# Supplementary figures and images for: First Investigation of Grass Carp Reovirus (GCRV) Infection in Amphioxus: Insights into Pathological Effects, Transmission, and Transcriptomic Responses
Source: Viruses. 2025 Oct 13;17(10):1367. doi: 10.3390/v17101367 (PMC12567996; doi:10.3390/v17101367)

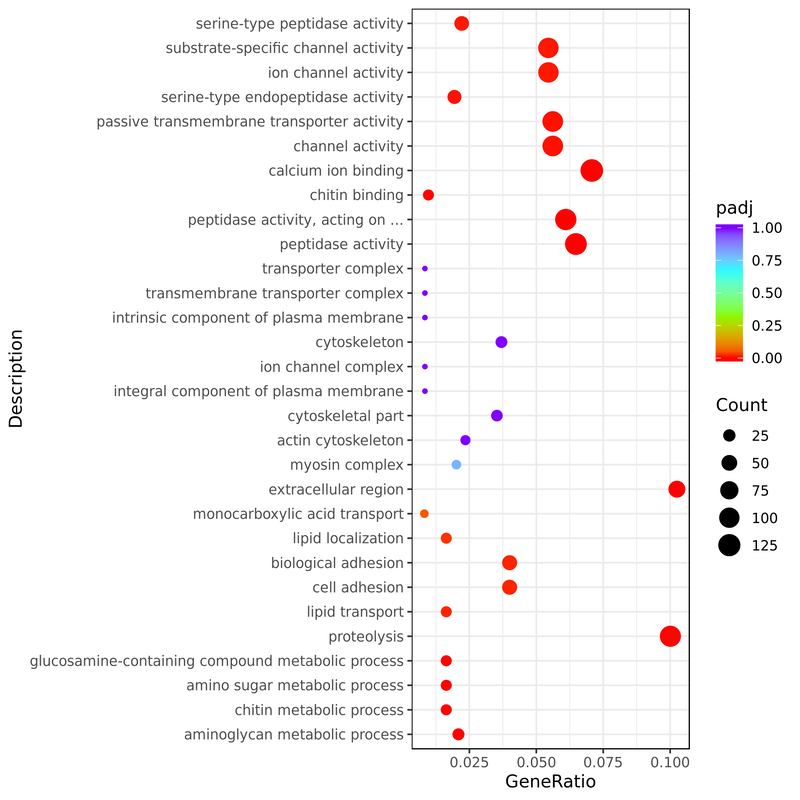

Supplement: Supplementary file 1 [file viruses-17-01367-s001.zip › Figure S1 GO.dot.png]
